# Supplementary material for: Elemental partitioning-mediated crystalline-to-amorphous phase transformation under quasi-static deformation
Source: Nat Commun. 2024 Feb 9;15:1223. doi: 10.1038/s41467-024-45513-7 (PMC10858257; doi:10.1038/s41467-024-45513-7)
Supplement: Supplementary file 2 — Supplementary Information [file 41467_2024_45513_MOESM2_ESM.pdf]

Supplementary Information for Elemental partitioning-mediated crystalline-to-amorphous phase transformation under quasi-static deformation

Ge Wu[1]*, Chang Liu[2], Yong-Qiang Yan[1], Sida Liu[3], Xinyu Ma[3], Shengying Yue[3], Zhi-Wei Shan[1]*

1. *Center for Advancing Materials Performance from the Nanoscale (CAMP-Nano) and Hysitron Applied Research Center in China (HARCC), State Key Laboratory for Mechanical Behavior of Materials, Xi'an Jiaotong University, 710049 Xi'an, China*
2. *Center for Alloy Innovation and Design (CAID), State Key Laboratory for Mechanical Behavior of Materials, Xi'an Jiaotong University, 710049 Xi'an, China*
3. *Laboratory for multiscale mechanics and medical science, SV LAB, School of Aerospace, Xi'an Jiaotong University, Xi'an 710049, China*

Email: gewuxjtu@xjtu.edu.cn (G. Wu); zwshan@xjtu.edu.cn (Z.-W. Shan)

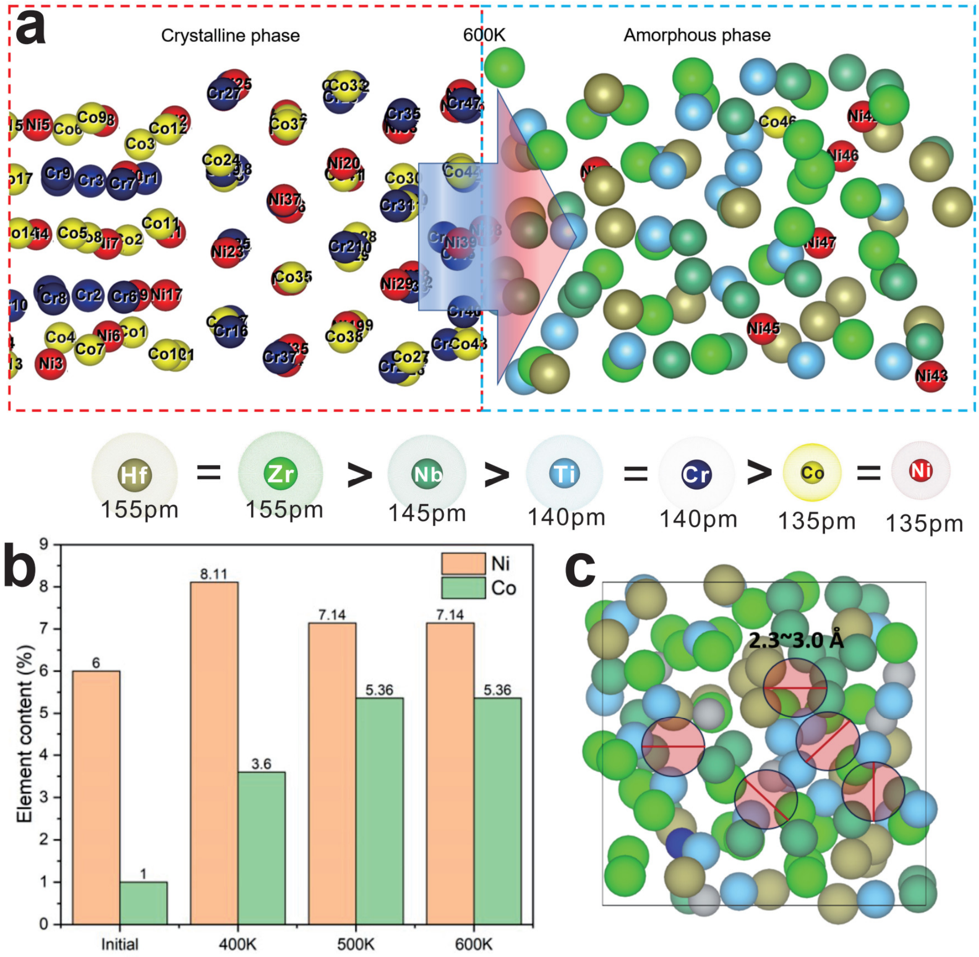

Supplementary Fig. 1 | Diffusion behavior of the crystal ($Cr_{35}Ni_{33}Co_{32}$ (at.%))-glass ($Zr_{28}Ti_{24}Nb_{22}Hf_{19}Ni_{6}Co_{1}$ (at.%)) system upon heating. a, the crystalline-amorphous model by ab initio molecular dynamic (AIMD) simulation at 600 K. **b,** The atomic percentage of Co and Ni in the amorphous phase during heating. **c,** Schematic diagram for the diffusion mechanism of Ni and Co, including the empirical atomic radius of each element and the interspace among the atoms in the amorphous phase.

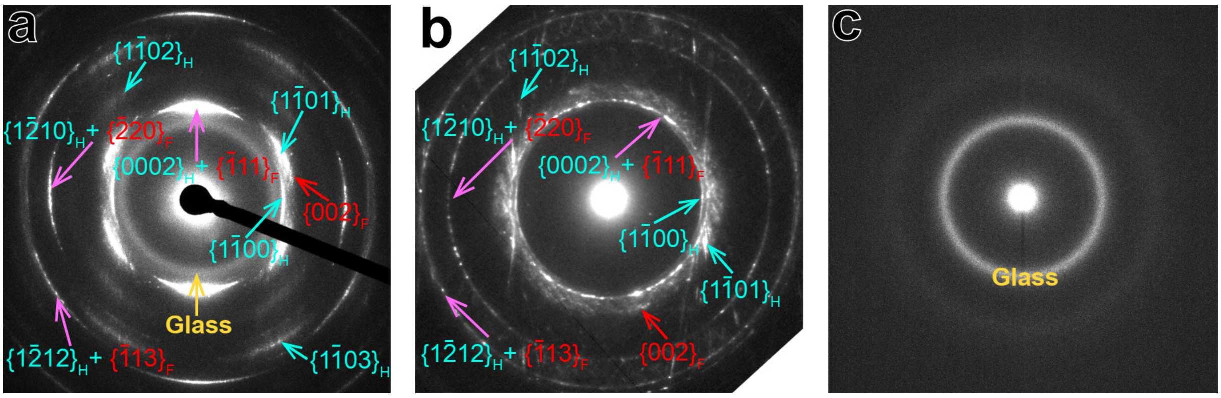

Supplementary Fig. 2 | Electron diffraction patterns of a, Crystal-glass nanolaminated alloy, indexed by an amorphous halo ring and crystalline FCC/HCP planes. **b,** Crystalline CrCoNi alloy, indexed by crystalline FCC/HCP planes. **c,** Amorphous TiZrNbHf-Cr-Co-Ni alloy. The experiments were conducted on cross-sectional TEM samples with identical conditions.

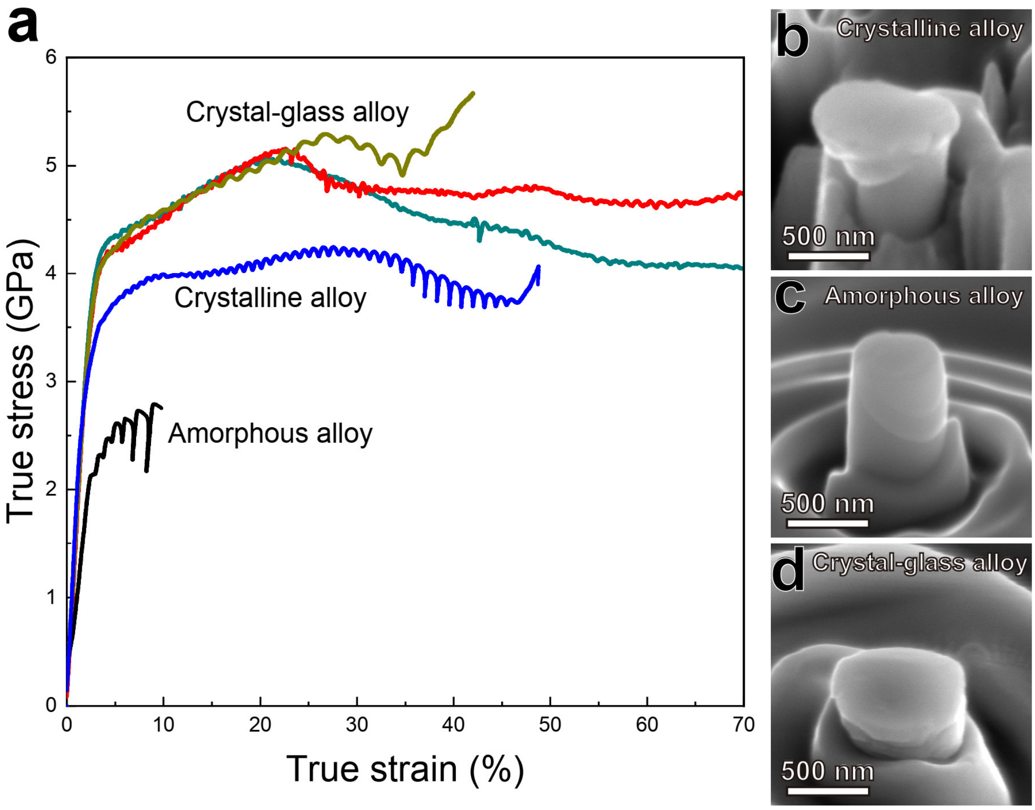

Supplementary Fig. 3 | Deformation behavior of the crystal-glass nanolaminated alloy. a, True stress-strain curves of the pillar samples tested with identical conditions at room temperature. **b-d,** SEM images of the corresponding deformed samples.

Supplementary Table 1 | The effective atomic velocities and the empirical atomic radius of each elements for the crystal ($Cr_{35}Ni_{33}Co_{32}$ (at.%))-glass ($Zr_{28}Ti_{24}Nb_{22}Hf_{19}Ni_6Co_1$ (at.%)) system.

Elements	Co	Ni	Cr	Ti	Zr	Nb	Hf
**Atomic velocity (m/s) at 600K**	503	504	536	559	405	401	289
**Empirical atomic radius (pm)**	135	135	140	140	155	145	155
